# Diagnostic potential of serum humanin in breast cancer among the Egyptian population

**DOI:** 10.1007/s12672-025-04325-x

**Published:** 2026-01-10

**Authors:** Maha H. Mohamed, Walaa Talaat Kamel, Eman H. Ibrahim, Ahmed Makboul, Amany Nasr Elemary, Doaa A. Mohammed

**Affiliations:** 1 Clinical Pathology Department, South Egypt Cancer Institute, Assiut University, Assiut, 71515 Egypt; 2 Medical Oncology and Hematological Malignancies Department, South Egypt Cancer Institute, Assiut University, Assiut, 71515 Egypt

**Keywords:** Breast cancer, Humanin, Mitochondrial-derived peptides, Biomarker

## Abstract

**Background:**

Globally, breast cancer is the most frequently diagnosed malignancy and the second leading cause of cancer-related mortality among women. The ongoing pursuit of early detection has driven interest in identifying and validating novel diagnostic biomarkers that could enhance prognosis and therapeutic outcomes. Humanin, a mitochondrial-derived peptide (MDP) with reported cytoprotective properties, has been implicated in cancer biology and may play a role in breast cancer pathogenesis.

**Methods:**

A total of 75 female patients with primary non-metastatic breast cancer and 70 age-matched healthy controls of comparable age were enrolled in this study. Serum concentrations of humanin were quantified using an enzyme-linked immunosorbent assay (ELISA).

**Results:**

Serum humanin concentrations were significantly elevated in breast cancer patients compared with healthy controls (*p* < 0.001). Receiver operating characteristic (ROC) curve analysis demonstrated that humanin effectively differentiated breast cancer patients from controls, with a sensitivity of 78.7% and specificity of 62.9%.

**Conclusion:**

These findings suggest that humanin could serve as a promising biomarker for breast cancer screening and early detection. Further large-scale studies are warranted to validate its diagnostic potential and establish its clinical utility.

## Introduction

Breast cancer remains the leading cause of cancer-related mortality among women worldwide [1]. Mammography continues to be the cornerstone of breast cancer detection and has contributed to reduced mortality rates [2]. However, this approach has its limitations, as false-positive findings and over-diagnosis of indolent lesions have raised considerable concern [3]. Consequently, there is a growing necessity for early, reliable biomarkers capable of predicting disease outcomes and tailoring treatment decisions [1]. In clinical practice, breast cancer biomarkers are primarily used to predict therapeutic response, monitor patients following primary treatment, and provide prognostic insights [4].

Mitochondria play fundamental roles in cellular energy metabolism, reactive oxygen species (ROS) generation, and regulation of cell survival—processes intricately linked to cancer development [5]. The discovery of mitochondrial-derived peptides (MDPs) has expanded the understanding of mitochondrial function beyond energy production [6]. This family comprises humanin, six Small Humanin-Like Peptides (SHLPs 1–6), and the Mitochondrial Open Reading Frame of the 12 S rRNA Type-c (MOTS-c) [7]. MDPs, encoded by mitochondrial DNA (mtDNA), act as hormone-like signaling molecules that influence cell proliferation and survival. Among them, Humanin has emerged as a critical regulator that safeguards cells against various stress-induced apoptotic signals [8].

Humanin is a short peptide of 24 amino acids encoded by a small open reading frame (sORF) within the 16 S rRNA region of mtDNA [9]. It can be translated in both the mitochondrial matrix and cytosol, producing functional 21- and 24-amino acid peptides, respectively [10]. Functionally, humanin modulates the mitochondrial apoptotic pathway through interactions with BCL-2 family proteins [11].

Humanin is expressed in diverse tissue types, such as the heart, liver, kidneys, testes, skeletal muscle, and brain [12]. Its cytoprotective and anti-apoptotic effects have been demonstrated across several species—humans, rats, and mice—and it has been proposed as a therapeutic target in various diseases such as Alzheimer’s disease, diabetes, and atherosclerosis [11, 13]. Humanin was originally identified during investigations into neuroprotective factors in unaffected brain regions of patients with Alzheimer’s disease [14].

Previous studies have also proposed a potential role for humanin in tumorigenesis. Elevated humanin expression has been observed in several cancers, including gastric carcinoma [15], bladder cancer [16], and pituitary tumors [17]. Furthermore, experimental data suggest that humanin expression is lower in females than in males and can be downregulated by estrogens in pituitary cells, indicating that gonadal steroids may influence its expression. This sex-related difference is believed to arise from the inhibitory effect of estrogens, particularly estradiol [18].

Despite being proposed as a potential oncopeptide nearly two decades ago, the precise role of humanin in cancer biology and therapy remains incompletely understood [1]. Given its potent anti-apoptotic activity, humanin has been hypothesized to facilitate tumor cell survival and thereby contributing to breast cancer progression [19]. Supporting this hypothesis, humanin has been detected in the serum of patients with cutaneous T-cell lymphoma but not in healthy individuals [20], and it appears to confer protection against chemotherapy-induced cytotoxicity [21].

Breast cancer, though extensively studied, still lacks an ideal biomarker for early and accurate diagnosis [22]. Mitochondrial metabolism has been implicated in breast cancer cell migration, invasion, and metastasis [23]. Interestingly, humanin expression has been reported to be upregulated in breast cancer biopsies compared with normal breast tissues [14]. Nevertheless, its diagnostic and prognostic relevance in breast cancer remains poorly understood. Given the persistent need for non-invasive, sensitive, specific, and cost-effective diagnostic tools, this study aims to evaluate whether serum humanin concentrations could serve as a reliable biomarker for early breast cancer detection in Egyptian female patients, potentially improving diagnostic precision and clinical management.

## Materials and methods

### Sample size calculation

The sample size for this study was determined by G-Power software, version 3.1.9.2, targeting the primary outcome of detecting a difference in the median serum humanin levels between patient and control groups. An independent samples t-test was selected, with parameters set as alpha (α) = 0.05, power = 0.95, and an anticipated medium effect size (d = 0.6). This yielded the minimum required sample of 122 subjects.

### Patients

This case–control study included a total of 145 female participants: 75 patients newly diagnosed with non-metastatic breast cancer and 70 healthy volunteers with no history of malignancy. The study was conducted at South Egypt Cancer Institute, Assiut University, between 2024 and 2025. The exclusion criteria for this study included pregnant or lactating female patients, serious concomitant co-morbidity that compromise safety of patients and double malignancy other than bilateral breast cancer. A written informed consent was obtained from all individual participants included in the study.

### Ethical considerations

The study protocol was reviewed and approved by the Institutional Review Board (IRB) of the South Egypt Cancer Institute, Assiut University (SECI-IRB IORG0006563; Approval No. 723). The study procedures adhered to the ethical principles outlined in the Declaration of Helsinki.

### Sample analysis

Venous blood samples were collected from all enrolled patients and control participants. Samples were centrifuged at 10,000 rpm for 10 min, and the separated serum was stored at − 80 °C until further analysis. Serum Humanin concentrations were quantified using a human Humanin (MT-RNR2) enzyme-linked immunosorbent assay (ELISA) kit (Fine Biotech Co. Ltd., Wuhan, China - Catalogue No. EH9261) following the manufacturer’s instructions. The assay has an analytical sensitivity of 0.12 pg/mL, with a detection range of 0.146–600 pg/mL. According to the validation data, intra-assay coefficients of variation (%CV) for low, medium, and high concentrations are 5.32%, 5.20%, and 4.54%, respectively. The inter-assay %CV for low, medium, and high concentrations are 5.1%, 5.05%, and 4.99%, respectively. All specimens were analyzed in duplicate, and results outside the linear range were repeated.

### Immunohistochemistry (IHC) and molecular subtyping

Estrogen receptor (ER), progesterone receptor (PR), and human epidermal growth factor receptor 2 (HER2) status were evaluated by pathologists in the Oncologic Pathology Department at our institution. Tissue samples were fixed in 10% formalin, embedded in paraffin, and processed using standard histopathological techniques. Section 5 μm thick were mounted on glass slides and stained with hematoxylin and eosin (H&E). Samples with 1% − 100% of tumor nuclei positive for ER or PR were interpreted as positive. For reporting of ER (not PR), if 1–10% of tumor cell nuclei were immunoreactive, specimens were reported as ER Low Positive [24]. HER2 status was scored on a 0–3 + scale based on staining intensity and completeness. Scores of 0 (no or faint incomplete staining in ≤ 10% of cells) and 1+ (faint incomplete staining in > 10% of cells) were classified as negative. A 2 + score indicated weak to moderate complete membranous staining in > 10% of tumor cells and was considered equivocal, warranting confirmatory testing by in situ hybridization (ISH) for HER2 gene amplification [25]. A 3 + score represented strong, complete, circumferential membranous staining in > 10% of tumor cells and was classified as positive. The Ki-67 proliferation index was determined manually by calculating the percentage of positively stained nuclei among 1000 counted tumor cells.

### Statistical analysis

Statistical analyses were performed using the Statistical Package for the Social Sciences (SPSS), version 27 (SPSS Inc., Chicago, IL, USA). The Shapiro–Wilk test was applied to assess data normality. Quantitative variables were expressed as mean ± standard deviation (SD) or median (interquartile range), depending on data distribution. Qualitative variables were summarized as frequencies and percentages. Comparisons between quantitative variables were made using the independent-samples *t*-test or one-way ANOVA for normally distributed data, and the Mann–Whitney *U* test for non-normally distributed data. Categorical variables were compared using the Chi-square (χ²) test. A *p*-value < 0.05 was considered statistically significant.

Diagnostic performance of serum humanin concentration was evaluated using receiver operating characteristic (ROC) curve analysis to determine the optimal cut-off value, as well as corresponding sensitivity and specificity for breast cancer detection.

## Results

### Demographic comparison of breast cancer and control groups

The mean age of breast cancer patients was 51.2 ± 11.8 years, while that of the control group was 50.5 ± 11.5 years. There was no statistically significant difference between the two groups regarding age or body mass index (BMI) (Table 1).

### Comparison of serum humanin concentrations between breast cancer patients and control group

**Fig. 1:**
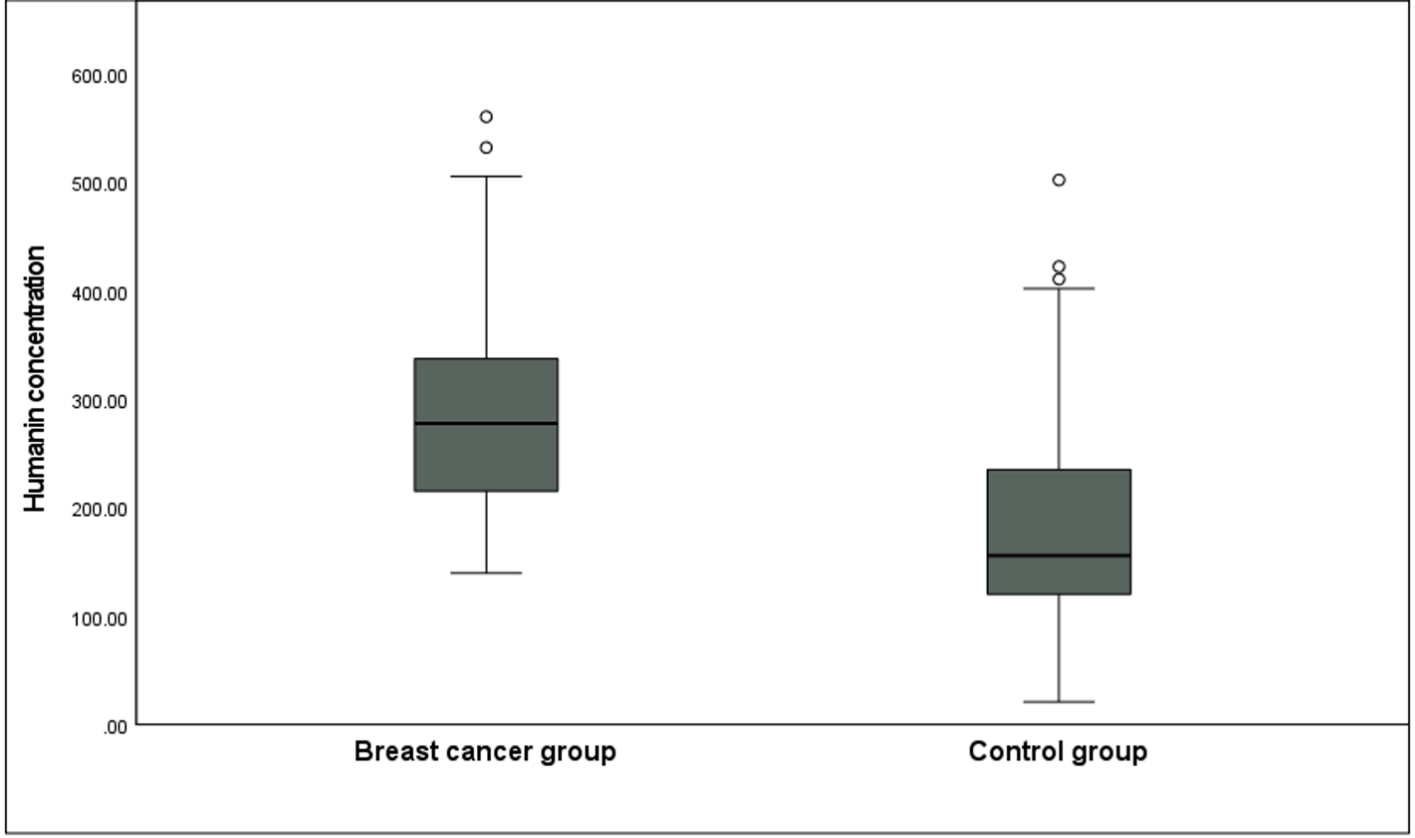
Box plot of serum humanin concentration in breast cancer patients and controls. Median values are denoted. humanin concentrations were significantly higher in breast cancer patients than in controls (*p* < 0.001)

Serum humanin concentrations were determined in both breast cancer patients and healthy controls. The median serum humanin concentration was significantly higher in breast cancer patients compared with the control group (277.1 pg/ml [205.7–336.8] vs. 155.5 pg/ml [118.5–235.5], *p* < 0.001) (Table 2; Fig. 1).

### Receiver operating characteristics (ROC) curve analysis for serum humanin concentration in breast cancer

**Fig. 2:**
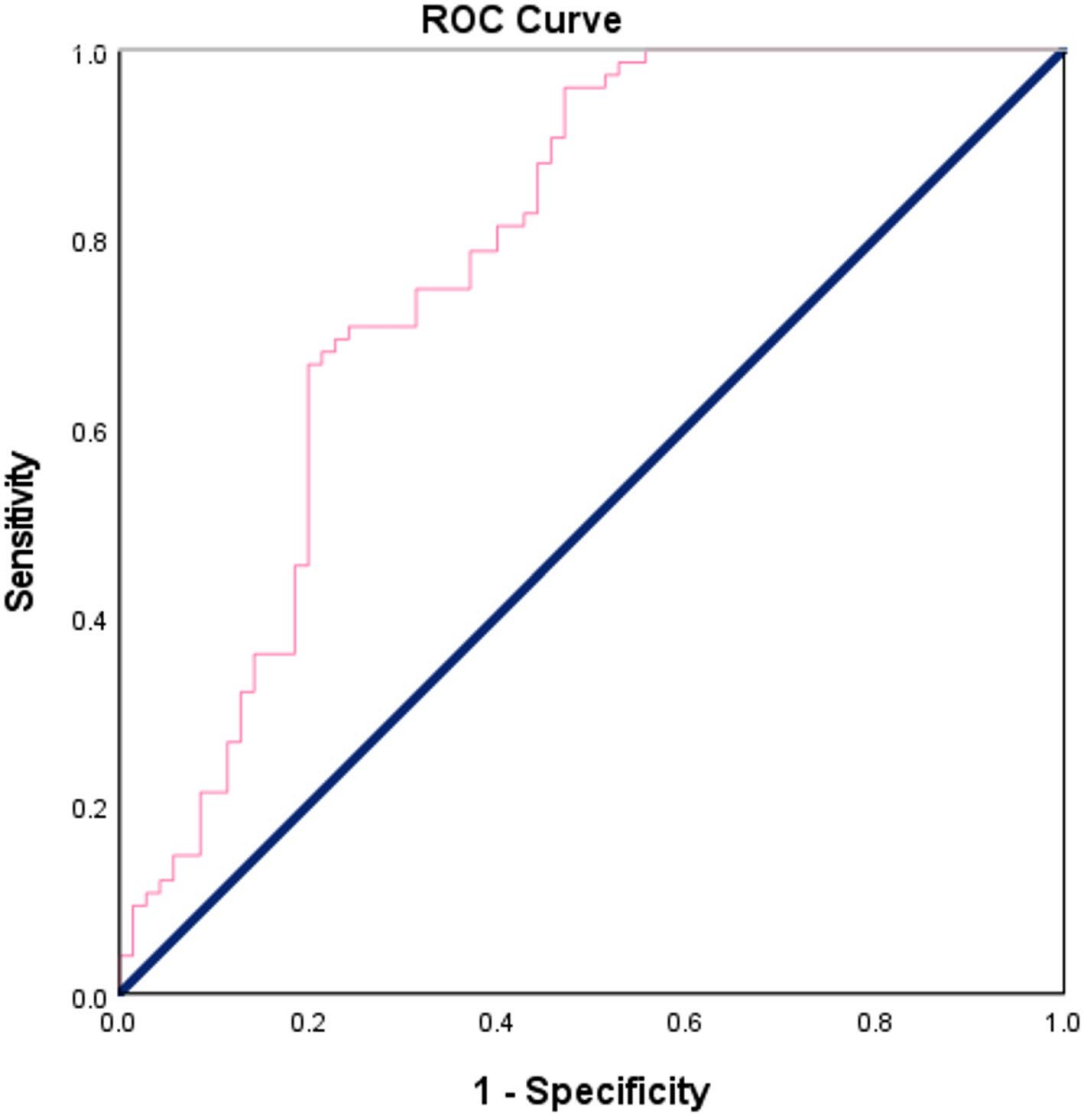
ROC curve of humanin concentration for discriminating between breast cancer patients and control group. The analysis yielded an AUC of 0.777 (*p* < 0.001), indicating good diagnostic performance. At a cut-off point of 205.69 pg/mL, humanin concentration showed a sensitivity of 78.7% and a specificity of 62.9%

Receiver operating characteristic (ROC) curve analysis demonstrated that serum humanin concentration could effectively differentiate breast cancer patients from healthy controls, yielding an area under the curve (AUC) of 0.777 (*p* < 0.001), which reflects good diagnostic performance. At an optimal cut-off value of 205.69 pg/mL, humanin concentration showed a sensitivity of 78.7% and a specificity of 62.9% (Table 3; Fig. 2).

### Associations between serum humanin concentration and clinicopathological features

The relationship between serum humanin concentration and baseline tumor characteristics, as well as treatment modalities, was evaluated among breast cancer patients. A significant association was found between serum humanin concentration and HER2 amplification status assessed by ISH (*p* = 0.013), as well as with the type of adjuvant chemotherapy regimen administered (*p* = 0.005). However, no significant associations were observed between humanin concentration and other tumor characteristics or treatment parameters, including tumor stage, grade, hormone receptor status, or the use of neoadjuvant therapy (Table 4).

## Discussion

Beyond their role in energy production, mitochondria are key contributors to tumorigenesis, as they participate in bioenergetic, biosynthetic, and signaling pathways that enable cancer cells to adapt to their microenvironment. Disturbances in cellular energy metabolism arising from mitochondrial DNA mutations, enzymatic defects, or general mitochondrial dysfunction are recognized hallmarks of cancer [14].

The human mitochondrial genome contains numerous sORFs that encode microproteins known as MDPs. These peptides function both within cells and in the systemic circulation, influencing a variety of physiological processes [26]. Among them, humanin, a peptide encoded by mitochondrial DNA, exerts strong cytoprotective effects, maintaining mitochondrial integrity and promoting cell survival under stress and aging conditions. Due to these properties, Humanin has been proposed as a potential therapeutic molecule in combating age-related disorders such as cardiovascular disease, neurodegeneration, and cancer [12].

Although the precise role of humanin in tumorigenesis remains incompletely understood, several studies have investigated its potential involvement in cancer development and progression. In the present study, serum humanin concentrations were significantly higher in patients with breast cancer compared to healthy controls. Using a cut-off value of 205.69 pg/mL, the test demonstrated a sensitivity of 78.7%, and specificity of 62.9%. These findings suggest that elevated serum humanin concentrations may serve as a promising biomarker for the early detection of breast cancer. Our results are consistent with those of Hekim et al., who also reported significantly higher serum humanin concentrations in breast cancer patients relative to controls, with a cut-off value of 254.4 pg/mL and corresponding sensitivity and specificity of 62.5% and 77.5%, respectively. Their study further supports the diagnostic potential of circulating humanin in breast cancer [14]. Experimental data by Moreno Ayala et al. also reinforce these observations at the tissue level. They demonstrated that humanin expression was markedly increased in malignant breast tissues compared with normal counterparts, particularly in triple-negative breast cancer (TNBC). Moreover, both humanin and its receptors were found to be upregulated in TNBC, where exogenous and endogenous humanin appeared to protect neoplastic cells against cytotoxic and anti-proliferative stimuli. Notably, administration of humanin promoted tumor progression and chemoresistance in experimental TNBC models [1]. Collectively, these findings highlight the complex and context-dependent role of humanin in breast cancer, acting potentially as both a biomarker of disease presence and a modulator of tumor biology. Our results also agree with previous studies showing that serum-based biomarker panels improve breast cancer detection and prognostication. Ma et al. demonstrated that the serum-based surface-enhanced Raman scattering (SERS) profiling can effectively discriminate early breast cancer patients from healthy controls using a composite silver-nanoparticle PSi Bragg reflector substrate. Their findings highlight the potential of serum proteomic signatures as a promising tool for early breast cancer detection [27].

The ROC curve analysis in the present study demonstrated an AUC of 0.777 which indicates a moderate diagnostic accuracy of serum humanin levels as a biomarker in distinguishing breast cancer patients from healthy controls. This performance is comparable to previous reports showing that single circulating biomarkers often achieve only modest accuracy due to limited sensitivity and specificity. Such findings support the growing evidence that relying on individual biomarkers may not provide sufficient diagnostic power, and that combining multiple markers into a biomarker panel can substantially enhance clinical utility. Although Abdulkarim et al. investigated mechanistic pathways in TNBC rather than serum biomarkers, their findings underscore the complexity of cancer biology and the limitations of relying on single molecular indicators [28]. Therefore, although humanin shows promising diagnostic potential in breast cancer detection, its integration into a broader biomarker panel may offer improved performance for future clinical applications.

In the present study, serum humanin concentration showed no significant association with key baseline tumor characteristics, including tumor stage, histological grade, lymphovascular invasion (LVI), or perineural invasion (PNI). This observation aligns with the findings of Hekim et al., who similarly reported no significant correlations between serum humanin concentrations and tumor parameters such as stage or grade. Moreover, consistent with their results, no associations were observed between humanin concentrations and metabolic or biochemical factors, including dyslipidemia and conventional tumor biomarkers such as CEA, CA15-3, and CA125 [14]. A study by Hui et al. demonstrated in a mouse breast cancer model that geraniin treatment—an ellagitannin present in many seeds, nuts, fruits, and plants—induced changes in multiple plasma proteins independent of tumor induction, suggesting that circulating proteins may not directly reflect tumor burden [29]. Mechanistic studies, such as Zhou et al., have shown that intracellular proteins can influence tumor metabolism and progression independently of classical tumor features [30]. While this does not directly involve circulating biomarkers, it highlights that molecular changes in breast cancer may not always correlate with stage or receptor status. Similarly, our data showed that serum humanin concentrations did not correlate with tumor stage, supporting the potential utility of serum humanin as an independent biomarker.

Serum humanin concentrations also showed no significant association with hormone receptor status. The hormonal regulation of humanin provides additional biological context for our findings. Previous studies have demonstrated that in experimental animals, humanin expression is influenced by sex hormones, particularly estrogen [18], suggesting that circulating levels may be modulated by hormonal status in women. This sex-specific regulation may contribute to interpatient variability in serum humanin and could partly explain the absence of correlation with hormone receptor status and tumor stage in our cohort. Future studies incorporating menopausal status, hormonal therapy exposure, and endocrine profiles may clarify the extent to which estrogen-mediated regulation could affect the diagnostic performance of humanin in breast cancer.

Interestingly, our analysis revealed a potential association between serum humanin concentration and HER2 amplification status as determined by ISH (*p* = 0.013). This finding may indicate a possible link between humanin expression and specific molecular subtypes of breast cancer, particularly those driven by HER2-related oncogenic pathways. In contrast, Hekim et al., demonstrated a positive correlation between serum humanin levels and Ki-67 expression, suggesting that humanin may be more closely related to proliferative activity rather than receptor status [14]. Such discrepancies may reflect differences in patient ethnicity and tumor biology. Our findings may also be interpreted in the context of genetic heterogeneity in breast cancer. A study by Baráti et al. highlighted that genetic diversity may influence tumor biology and circulating biomarkers [31], suggesting that inter-individual differences in serum humanin concentrations could reflect underlying hereditary and molecular heterogeneity. These observations underscore the potential value of integrating biomarker assessment with genetic profiling to improve risk stratification and early detection strategies.

Furthermore, a significant association was observed between serum humanin concentrations and the type of adjuvant chemotherapy planned (*p* = 0.005). Although the mechanism underlying this relationship remains unclear, it could reflect indirect associations with molecular subtype distribution or treatment decision patterns rather than a direct biological interaction. While direct evidence in breast cancer is limited, recent studies in other malignancies highlight how molecular regulators can profoundly influence oncogenesis and treatment resistance. A study by Peng et al. demonstrated that proteasome subunit PSMD12 promotes hepatocellular carcinoma (HCC) progression via CDK1 stabilization [32]. Another study by Ning et al. showed that hepatitis B virus (HBV) mutations increase HCC risk by altering tumor suppressor gene mutation rates [33]. Ramalingam et al. reviewed the role of non-coding RNAs in HBV-related HCC [34]. These studies collectively underscore the broader principle that small regulatory molecules - whether proteasome subunits, viral mutations, non-coding RNAs, or peptides like humanin - can act as biomarkers and modulators of cancer biology. This conceptual parallel strengthens the translational relevance of our study, suggesting that humanin may similarly influence chemoresistance and therapeutic outcomes in aggressive breast cancer subtypes. Additional studies incorporating larger cohorts, longitudinal sampling, and functional analyses are warranted to clarify the potential role of humanin in tumor biology and treatment response.

The rapid evolution of breast cancer therapeutics increasingly incorporates nanotechnology and computational innovations to enhance treatment precision and efficacy. Recent advances, such as the development of reduction-responsive epirubicin nanoassemblies described by Yuan et al., illustrate how novel drug-delivery systems can improve potency while reducing systemic toxicities [35]. At the same time, AI-driven approaches, including those highlighted by Hussein et al., are transforming biomarker discovery and personalized treatment strategies, particularly in aggressive subtypes such as TNBC [36]. Within this expanding landscape, the identification of circulating mitochondrial peptides such as humanin gains added translational importance. Their stability and biological relevance raise the possibility that such peptides could be incorporated into next-generation therapeutic or diagnostic platforms, aligning with broader precision medicine trends.

The growing emphasis on personalized and precision oncology has heightened the need for reliable circulating biomarkers that can refine diagnosis, predict treatment response, and guide individualized therapeutic strategies. Recent work in precision-medicine research illustrates this shift. A study by Duman and Pichler emphasizes how advanced pre-clinical breast cancer models and migration platforms are being optimized to mirror patient-specific tumor behavior [37], supporting more tailored therapeutic development. Likewise, molecular studies such as that of Ramalingam et al., which identified RPS3 as a key biomarker of resistance to KRAS-targeted therapy in pancreatic ductal adenocarcinoma cells, highlight how biomarker discovery can directly inform treatment selection and overcome resistance mechanisms [38]. Within this evolving landscape, the characterization of MDPs such as humanin may provide additional opportunities for clinical decision support. Its measurable serum levels and associations with tumor biology suggest that it could contribute to future precision-medicine approaches by complementing existing molecular markers and helping refine individual management strategies in breast cancer.

Although our findings support the potential of serum humanin as a diagnostic biomarker, the cohort size and demographic homogeneity limit broader acceptability. Future studies across larger, multi-ethnic populations and multiple clinical centers are necessary to confirm robustness and enhance generalizability.

**Table 1:** Comparison of descriptive data between breast cancer and control groups

Parameter	Breast cancer group (*n* = 75) Mean ± SD	Control group (*n* = 70) Mean ± SD	*P* value*
Age (Years)	51.2 ± 11.8	50.5 ± 11.5	0.703
BMI (kg/m^2^)	29.48 ± 6.89	28.71 ± 6.17	0.479

BMI: Body mass index; SD: Standard deviation

*P value was significant if < 0.05

**Table 2:** Serum humanin concentration among the studied groups

Parameter	Breast cancer group (*n* = 75) Median (25th – 75th percentile)	Control group (*n* = 70) Median (25th – 75th percentile)	*P* value*
Humanin (pg/mL)	277.1 (205.7–336.8)	155.5 (118.5–235.5)	**< 0.001**

*P value was significant if < 0.05

**Table 3:** Diagnostic accuracy of serum humanin concentration among the studied groups

Sensitivity	78.7%	Accuracy	71.03%
Specificity	62.9%	Area Under Curve (AUC)	0.777
Cut-off	205.69 pg/mL	*P* value*	**< 0.001**

*P value is significant if < 0.05

**Table 4:** Associations between serum humanin concentration and clinicopathological features

Tumor characteristics		Number (%)	Humanin concentration (pg/mL)	*P* value*
Tumor stage	Stage 1	6 (8%)	239.3 ± 59.6	0.344
	Stage 2	18 (24%)	304.86 ± 100.45	
	Stage 3	51 (68%)	292.19 ± 196.04	
Tumor grade	Low grade	4 (5.3%)	287.97 ± 86.84	0.996
	High grade	68 (90.7%)	291.31 ± 97.37	
LVI	Present	54 (72%)	303.26 ± 90.65	0.121
	Absent	13 (17.3%)	243.01 ± 80.77	
	Not known	8 (10.7%)	286.26 ± 129.81	
PNI	Present	23 (30.7%)	308.79 ± 97.08	0.564
	Absent	44 (58.7%)	282.57 ± 88.15	
	Not known	8 (10.7%)	286.26 ± 129.81	
Estrogen	Positive	63 (84%)	288.07 ± 96.35	0.545
	Negative	12 (16%)	306.38 ± 90.99	
Progesterone	Positive	58 (77.3%)	286.25 ± 91.16	0.428
	Negative	17 (22.7%)	307.23 ± 109.14	
Ki-67	Low expression	5 (6.7%)	339.54 ± 146.43	0.24
	High expression	70 (93.3%)	287.54 ± 91.02	
HER2 by IHC	Negative	47 (62.7%)	295.09 ± 99	0.889
	Equivocal	13 (17.3%)	285.99 ± 110.22	
	Positive	15 (20%)	282.54 ± 71.3	
HER2 by ISH	Amplified	17 (22.7%)	289.98 ± 70.43	**0.013**
	Not amplified	57 (76%)	286.6 ± 96.12	
	Not done yet	1 (1.3%)	559.54 ± 0	
Neoadjuvant chemotherapy	Yes	37 (49.3%)	289.53 ± 91.54	0.896
	No	38 (50.7%)	292.43 ± 99.75	
Type of neoadjuvant therapy	Anthracycline-based	5 (13.5%)	355.50 ± 97.85	0.173
	Anthracyclines, then taxenes	28 (75.7%)	283.67 ± 90.56	
	Taxenes	4 (10.8%)	248.09 ± 64.2	
Response to 1 st line	Complete remission	1 (4.8%)	168.63 ± 0	0.367
	Progressive disease	2 (9.5%)	273.88 ± 103.92	
	Partial response on neoadjuvant therapy	18 (85.7%)	316.93 ± 104.33	
Adjuvant chemotherapy	Yes	40 (76.9%)	297.25 ± 102.86	0.912
	No	12 (23.1%)	293.4 ± 114.94	
Adjuvant therapy plan	Anthracycline-based	8 (20%)	213.11 ± 64.75	**0.005**
	Anthracyclines, then taxenes	29 (72.5%)	307.86 ± 93.84	
	Taxenes	3 (7.5%)	419.07 ± 127.05	
Radiotherapy	Yes	23 (60.5%)	304.72 ± 102.72	0.488
	No	3 (7.9%)	240.84 ± 100.24	
	Not yet started	12 (31.6%)	271.01 ± 114.1	
Targeted Therapy	Yes	16 (21.3%)	288.3 ± 72.39	0.925
	No	59 (78.7%)	291.74 ± 100.97	
Anti-HER2	Trastuzumab	10 (62.5%)	305.55 ± 64.03	0.23
	Trastuzumab-Pertuzumab	6 (37.5%)	259.53 ± 82.16	
Zoladex	Yes	2 (2.7%)	403.71 ± 142.6	0.09
	No	73 (97.3%)	287.92 ± 93.06	

LVI: Lymphovascular invasion; PNI: Perineural invasion; HER2: Human epidermal growth factor receptor 2; IHC: Immunohistochemistry; ISH: In situ hybridization

*P value was significant if < 0.05

## Conclusion

Our findings suggest that serum humanin may serve as a valuable and promising biomarker for the early diagnosis of breast cancer. Although not yet integrated into routine clinical practice, its potential diagnostic utility could be significantly enhanced when combined with established tumor markers such as CA15-3 and CA125, as well as with advanced imaging modalities. This aligns with current proteomic and computational approaches that emphasize combining multiple indicators to improve diagnostic performance.

Advanced computational frameworks demonstrate how multi-source network analyses can identify prognostic and heterogeneous biomarkers with superior discriminative power [39]. Incorporating such methods in the future could enhance the predictive value of humanin as a diagnostic biomarker.

Furthermore, emerging diagnostic technologies—including activatable fluorescent probes such as those reviewed by Men X et al.—illustrate the growing potential of highly sensitive imaging modalities capable of detecting molecular events in vivo [40]. Comparing or eventually integrating humanin with such detection tools may enhance early diagnosis.

Finally, longitudinal evaluation of serum humanin concentrations in patients undergoing therapy could help establish its role as a monitoring tool for treatment response and disease recurrence.

Together, these directions define a clear pathway for refining and validating humanin as a clinically actionable biomarker within modern precision-oncology frameworks and algorithms for breast cancer management.

## Data Availability

No datasets were generated or analysed during the current study.

## Abbreviations

AUC: Area under the curve
BMI: Body mass index
CV: Coefficient of Variation
ELISA: Enzyme-linked immunosorbent assay
ER: Estrogen receptor
H&E: Hematoxylin and eosin
HBV: Hepatitis B Virus
HCC: Hepatocellular carcinoma
HER2: Human epidermal growth factor receptor 2
IHC: Immunohistochemistry
IRB: Institutional review board
ISH: In situ hybridization
LVI: Lymphovascular invasion
MDP: Mitochondrial-derived peptide
MOTS-c: Mitochondrial Open Reading Frame of the 12 S rRNA Type-c
mtDNA: Mitochondrial DNA
PNI: Perineural invasion
PR: Progesterone receptor
ROC: Receiver operating characteristic
ROS: Reactive oxygen species
SERS: Surface-enhanced Raman scattering
SHLPs: Small Humanin-Like Peptides
sORF: Small open reading frame
SPSS: Statistical Package for Social Sciences
TNBC: Triple negative breast cancer
